# Toward an Appropriate Classification of Adult-Onset Diabetes Mellitus: A Descriptive Study in Primary Care

**DOI:** 10.3390/jcm15218317

**Published:** 2026-10-26

**Authors:** Pilar Vich-Pérez, Belén Taulero-Escalera, Paula Regueiro-Toribio, Irene Moratinos-Recuenco, Javier Hernández-Jiménez, Laura Villanova-Cuadra, Ignacio Sevilla-Machuca, Julia Timoner-García, Mario Martínez-Grandmontagne, Tania Abós-Pueyo, Cristina Álvarez-Hernández-Cañizares, Germán Reviriego-Jaén, Alberto Serrano-López-Hazas, Inés Gala-Molina, Mar Sanz-Pascual, Miguel Angel Salinero-Fort, on behalf of the LADA-PC Consortium

**Affiliations:** 1 Los Alpes Health Center, 28022 Madrid, Spain; 2 Foundation for Biosanitary Research and Innovation in Primary Care, The Hospital La Paz Institute for Health Research (IdiPAZ), 28046 Madrid, Spain; 3 Department of Child and Adolescent Psychiatry, Institute of Psychiatry and Mental Health, Hospital General Universitario Gregorio Marañón, Gregorio Marañón Institute of Health Research(IiSGM), Biomedical Research Centre for Mental Health (CIBERSAM), Carlos III Health Institute (ISCIII), School of Medicine, Universidad Complutense de Madrid (UCM), 28040 Madrid, Spain; 4 Mar Báltico Health Center, 28033 Madrid, Spain; 5 Doctor Cirajas Health Center, 28017 Madrid, Spain; 6 Sanchinarro Health Center, 28050 Madrid, Spain; 7 Estrecho de Corea Health Center, 28027 Madrid, Spain; 8 Canillejas Health Center, 28022 Madrid, Spain; 9 Barajas Health Center, 28042 Madrid, Spain; 10 García Noblejas Health Center, 28037 Madrid, Spain; 11 Benita de Ávila Health Center, 28043 Madrid, Spain; 12 Aquitania Health Center, 28032 Madrid, Spain; 13 Alfonso X El Sabio University, 28691 Madrid, Spain; 14 Research Network on Chronicity, Primary Care and Health Promotion—RICAPPS—(RICORS), 28029 Madrid, Spain; 15 General Directorate of Research and Documentation, Department of Health, 28013 Madrid, Spain

**Keywords:** adult-onset diabetes, autoimmune diabetes, latent autoimmune diabetes in adults (LADA), primary care, cross-sectional study

## Abstract

**Background:** Type 2 diabetes mellitus (T2DM) is the most common form of diabetes in adults, although autoimmune reactions against pancreatic islets may also occur, leading to early insulin dependence and specific clinical features. The misclassification of autoimmune diabetes (AD) in adults has clinical implications in primary care (PC), where autoantibody testing is limited. The aim of this study was to estimate the prevalence of AD in adults at the time of diagnosis and to characterize its clinical, metabolic and lifestyle profile in comparison with that of T2DM patients. **Methods:** A cross-sectional study was conducted at 12 PC centers in Madrid (Spain). A total of 1006 adults aged 30 years or older who had been diagnosed with diabetes within the previous four years were included. Autoantibodies against pancreatic islets (anti-GAD65 and anti-IA-2) were analyzed in a centralized laboratory. Clinical interviews, physical examinations and reviews of electronic medical records were carried out. **Results:** The prevalence of AD was 7.56%. Patients with AD were younger and leaner and had more hyperglycemic symptoms and higher HbA1c levels. They had higher HDL cholesterol levels, lower triglyceride levels and more favorable cardiometabolic indices than patients with T2DM did. Patients with AD with LADA criteria differed from those with early insulin dependence. **Conclusions:** Autoimmune diabetes is a relatively common form of diabetes in adults and presents a clinical and metabolic profile that differs from that of T2DM. Improved recognition and targeted screening in primary care may facilitate accurate diagnosis and more appropriate therapeutic decision-making.

## 1. Introduction

Adult-onset diabetes mellitus (DM) is usually diagnosed and classified as type 2 diabetes (T2DM), in which insulin resistance is the primary pathogenic mechanism. However, a subgroup of newly diagnosed adults with diabetes exhibit autoimmunity against the insulin-producing cells of the pancreatic islets, resulting in an earlier insulin requirement than expected in patients with T2DM. Despite its importance, adult-onset autoimmune diabetes is often underdiagnosed or misclassified as T2DM, which has clinical and prognostic implications for patients. Accurate diagnosis and classification of adult-onset diabetes mellitus is crucial, given the reported prevalence of autoimmune diabetes in various studies (2–12%) and the negative consequences of misclassification [1].

Currently, there is no universally accepted consensus regarding the terminology and classification of adult-onset diabetes with pancreatic autoimmunity.

Latent autoimmune diabetes of the adult (LADA) is frequently described as a hybrid phenotype characterized by autoantibodies against pancreatic islets and initial insulin independence in individuals over 30 years of age. However, the distinction between LADA and classic type 1 diabetes (T1DM) in adults is controversial. Some experts highlight the gradual progression to insulin dependence and the presence of phenotypic features that overlap with type 2 diabetes as justification for differentiating LADA, whereas others consider it part of a continuum within T1DM [2].

This lack of consensus on classification criteria complicates diagnosis, clinical trial design, and therapeutic decisions. The American Diabetes Association (ADA) currently includes LADA in patients with T1DM and specifies that the presence of islet autoantibodies in the context of confirmed DM is sufficient for diagnosis [3]. Therefore, current classification challenges underscore the need for more precise diagnostic frameworks that incorporate both immunological and clinical phenotypes.

The heterogeneity and variability of autoimmune markers further complicate their classification. Autoantibodies can be of diverse types (anti-insulin autoantibodies (IAA), anti-glutamic acid decarboxylase antibodies (GADA), anti-IA 2 antibodies (IA 2A), and anti-zinc transporter 8 antibodies (ZnT8A)), and they also show quantitative differences in terms of titer and number of antibodies present.

Among adults with LADA, anti-GAD65 antibodies are the most frequently detected [4,5,6]. On the other hand, some individuals may test positive for these autoantibodies without developing diabetes [7], and these autoantibodies are frequently found in the relatives of patients with type 1 diabetes mellitus (T1DM) [8]. Additionally, antibody titers tend to be higher in the early stages of DM1 and may decrease over time, and high titers and the presence of multiple autoantibodies are associated with earlier insulin dependence [9,10].

To address this variability, the ADA diagnostic algorithm incorporates C-peptide measurement in selected cases, given that values below 200 pmol/L support a diagnosis of T1DM even in the absence of demonstrable autoimmunity [11].

The prevalence of autoimmune diseases, including T1DM, is increasing in many countries, underscoring the importance of accurate classification at the time of diagnosis [2]. In Spain, most people with diabetes are diagnosed and treated in primary care settings. In several autonomous communities, including Madrid, primary care physicians do not have access to tests for autoantibodies against pancreatic islet cells or C-peptide, which limits the accuracy of diagnosis. For this and other reasons (such as a lack of diagnostic awareness among healthcare professionals regarding this subtype of diabetes), knowledge of the prevalence and clinical profile of adult-onset autoimmune diabetes in primary care settings remains limited.

In summary, the aim of this study was to determine the prevalence of autoimmune diabetes among adults at the time of disease onset in primary care, to describe its clinical and metabolic characteristics and to compare these characteristics with those of individuals who did not present with autoimmune markers. The ultimate aim is to improve diagnostic classification and facilitate more appropriate therapeutic decision-making.

## 2. Materials and Methods

### 2.1. Study Design and Setting

This multicenter, observational, descriptive, and cross-sectional study was conducted at primary care centers in the Community of Madrid, Spain. The study involved 12 urban health centers with a centralized reference laboratory at the Ramón y Cajal University Hospital.

### 2.2. Participants and Eligibility

The study population consisted of adults aged 30 years or older with a diagnosis of diabetes mellitus (DM) recorded in their electronic medical records (EMR). Eligible participants were those who had been diagnosed within the previous four years and who had a minimum disease duration of at least six months.

The exclusion criteria included gestational diabetes, homebound status and physical or mental disabilities that prevented completion of the interview or examination.

A total of 1006 participants with available pancreatic islet autoantibody data constituted the final sample for the primary analysis. Recruitment was conducted through consecutive/convenience sampling by 184 healthcare professionals (family physicians and nurses). Although the study received funding and ethics approval in 2019, participant enrolment commenced on 30 March 2022 after completion of the logistical, administrative and organisational procedures required for study implementation. The study was conducted between 30 March 2022 and 7 June 2024, corresponding to the enrolment of the first and last participants, respectively.

### 2.3. Data Collection and Laboratory Measurements

Data were gathered through clinical interviews, physical examinations, EMR reviews, and laboratory testing.

Anti-GAD and anti-IA-2 autoantibodies were analyzed at the central laboratory of Hospital Ramón y Cajal and quantified using commercially available ELISA kits (RSR Limited, Cardiff, UK), according to the manufacturer’s instructions. The assays were processed using the EUROIMMUN Analyzer I platform (Euroimmun, Lübeck, Germany).

The results were classified as positive or negative according to the laboratory’s established cutoff values.

Other Laboratory Parameters: Fasting plasma glucose, HbA1c, and lipid profiles (total cholesterol, high-density lipoprotein (HDL) cholesterol, low-density lipoprotein (LDL) cholesterol, and triglyceride levels) were obtained. Renal status was assessed using the albumin-creatinine ratio (ACR) and EMR codes for chronic kidney disease.

### 2.4. Variables and Operational Definitions

Diabetes status was determined according to the diagnostic criteria of the American Diabetes Association (ADA). Autoimmune diabetes (AD) was defined as the presence of at least one pancreatic islet autoantibody, specifically anti-GAD65 or IA-2. Latent autoimmune diabetes in adults (LADA) was classified following the Immunology of Diabetes Society criteria: age > 30 years at onset, positivity for at least one islet autoantibody, and the absence of insulin requirements during the first six months after diagnosis.

Lifestyle habits were assessed using validated instruments. Physical activity was evaluated through a reduced version of the International Physical Activity Questionnaire (IPAQ). Adherence to the Mediterranean diet was measured using a modified 14-item Mediterranean Diet Adherence Screener (MEDAS), in which wine consumption was standardized to two glasses per week to improve the detection of alcohol-related risk.

Comorbidities, including hypertension, hypercholesterolemia, and metabolic syndrome, were defined according to internationally accepted clinical standards, specifically the National Cholesterol Education Program Adult Treatment Panel III (NCEP ATP III) criteria.

To further characterize the metabolic profile and estimate insulin resistance, three established cardiometabolic indices were calculated using validated formulas: the triglyceride–glucose index (TyG), the triglyceride–glucose–body mass index (TyG-BMI), and the triglyceride-to-HDL cholesterol ratio (TG/HDL-c). These indices provide complementary information on metabolic dysfunction and cardiometabolic risk beyond conventional biochemical measurements.

### 2.5. Statistical Analysis

Statistical analyses were performed using SPSS version 26.0 (IBM Corp. Armonk, NY, USA) and STATA version 16 (StataCorp, College Station, TX, USA). Quantitative variables are summarized as the mean and standard deviation (SD) or median and interquartile range (IQR), depending on their distribution. Categorical variables were described using absolute counts and relative frequencies.

Comparisons between subgroups (e.g., antibody-positive vs. antibody-negative participants) were conducted using Student’s *t*-test for normally distributed continuous variables and the Mann–Whitney U test for non-normally distributed data. Differences in proportions were assessed using the chi-square test.

### 2.6. Ethical Considerations

The study was approved by the Research and Medicines Ethics Committee of the Community of Madrid (code: JCAH/PVP/LADA/2019/1). All participants provided written informed consent. The study adhered to the Declaration of Helsinki and ensured data confidentiality in compliance with Spanish data protection laws (Organic Law 3/2018).

## 3. Results

**Figure 1:**
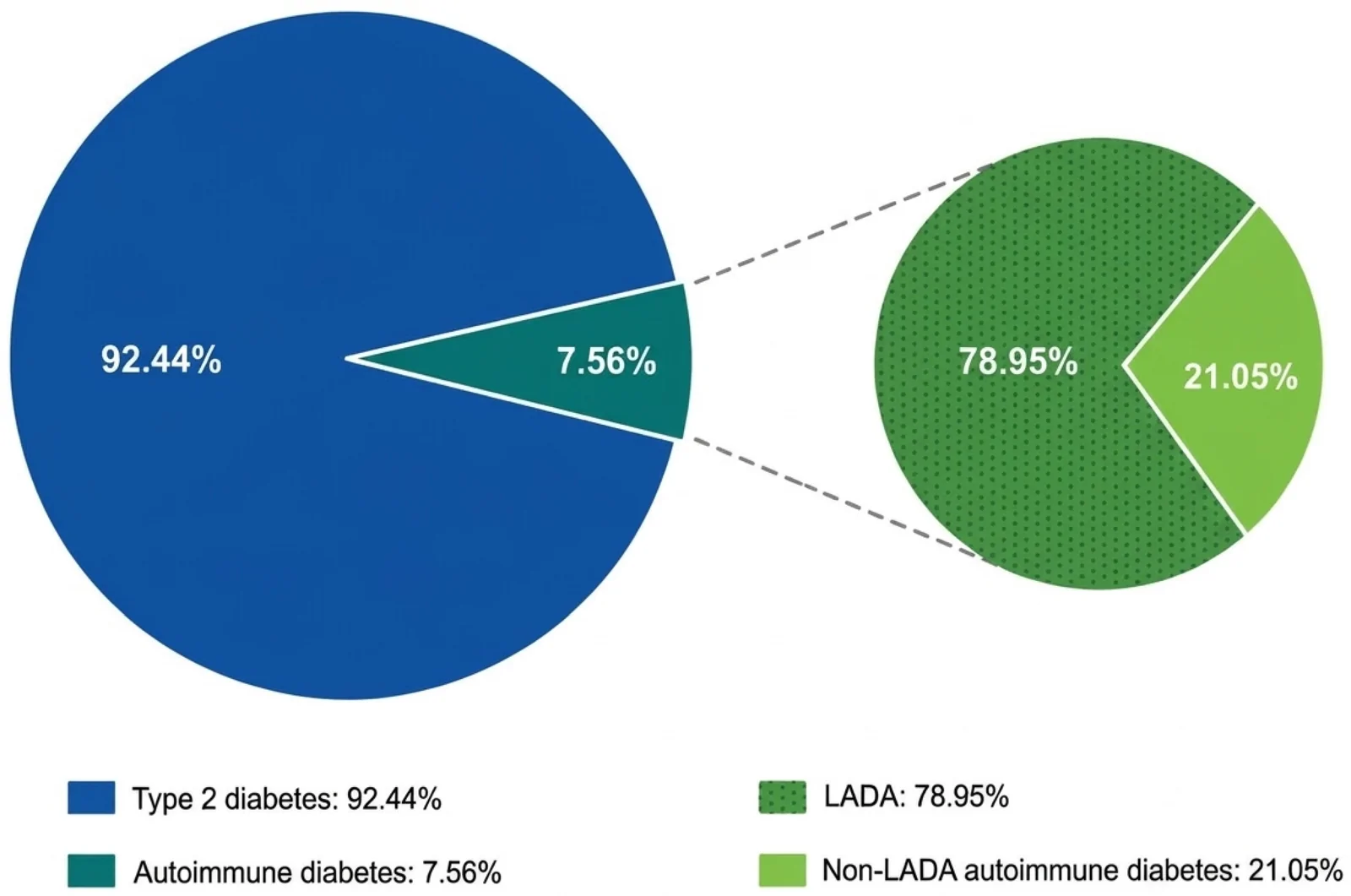
Distribution of patients with newly diagnosed diabetes mellitus according to the presence of antibodies against pancreatic islets and the LADA criteria.

The study included 1006 adult patients over the age of 30 with a recent diagnosis of diabetes mellitus from 12 primary care centers. Among these patients, 92.44% had T2DM, and 7.56% had an autoimmune form of the disease (Figure 1).

With respect to the characteristics of these patients, significant differences were found between AD patients and those with negative antibodies (T2DM). These differences included demographic and anthropometric characteristics, clinical presentation, perceived health, glycemic control at diagnosis, metabolic profiles, the presence of thyroid autoimmunity, other personal history of autoimmune conditions and certain comorbidities and medical history (Table 1).

Notable differences were observed in age, as patients with AD were generally younger—with a mean age of 59.2 years—than those who were antibody-negative—whose mean age was 62.6 years (*p* = 0.020); furthermore, a significantly greater percentage of antibody-positive patients was diagnosed before age 50 (27.6% versus 14.5%, *p* = 0.002).

With regard to body mass index (BMI), the AD group had a significantly lower mean BMI (28.3 kg/m^2^ compared to 30.9 kg/m^2^, *p* = 0.001 among patients with T2DM); notably, 34.7% of antibody-positive patients had a normal body mass index (<25 kg/m^2^), whereas only 11.7% of antibody-negative patients fell into this category (*p* < 0.001).

When we focused on symptoms, patients with positive antibodies were much more likely to have hyperglycemic symptoms at the time of diagnosis (39.5% vs. 17.3%, *p* < 0.001), and with regard to glycemic control at the time of diagnosis, the HbA1c level was significantly higher in patients with positive antibodies (8.4% vs. 7.7%, *p* = 0.015).

Furthermore, the perceived health status of patients with autoimmune diabetes was worse, as 6.6% of patients in this group rated it as “poor,” compared with only 1.6% in the T2DM group.

When the lipid and metabolic profiles of both groups were compared, higher levels of HDL cholesterol were detected in patients with autoimmune diabetes than in patients with T2DM (52.4 vs. 47.5 mg/dL, *p* = 0.001), and significantly lower triglyceride levels were detected in the group with antibodies (116 vs. 146.7 mg/dL, *p* = 0.008), and three lower metabolic indices were detected in patients with AD (triglycerides/HDL, triglycerides/glucose, and triglycerides/glucose/BMI; *p* < 0.05 in all cases).

Furthermore, both groups showed differences in comorbidities, with the T2DM group exhibiting a higher burden of cardiovascular and metabolic diseases, including a higher incidence of hypertension (57.4% in T2DM vs. 35.5% in AD, *p* < 0.001), hypercholesterolemia (67.1% in T2DM vs. 43.4% in AD, *p* < 0.001), and metabolic syndrome (60.4% in T2DM vs. 38.7% in AD, *p* < 0.001). Coronary artery disease was also more common in patients without antibodies (7.9% vs. 1.3%, *p* = 0.035).

Finally, patients who had AD had a higher prevalence of other autoimmune diseases (17.1% vs. 6.7%, *p* = 0.001), and the presence of antithyroid antibodies was also more common in this group (31,6% vs. 8%, *p* < 0.001 for TPO antibodies and 19,7% vs. 7,8%, *p* < 0.001 for Tg-Ab).

In summary, patients with positive antibodies tend to be younger, thinner, and more symptomatic at diagnosis and have higher HbA1c levels and a higher prevalence of associated autoimmune diseases, but they exhibit fewer typical features of metabolic syndrome (such as hypertension or obesity) than patients without antibodies do.

In addition, we subsequently analyzed the subgroup of patients with autoimmunity and the differences between the group of patients with anti-pancreatic islet antibodies who met the criteria for LADA, who were not insulin-dependent during the first six months after diagnosis (78.95% of patients with AD), and those who had antibodies but did not meet the criteria for LADA, who were insulin-dependent during the first six months after diagnosis (21.05% of patients with AD). The results are summarized in Table 2.

Age differences were also observed among patients with AD, as those with LADA were significantly older—with a mean age of 61.8 years—than the non-LADA AD group, whose mean age was 51.7 years. Furthermore, half of the patients with non-LADA AD were diagnosed before age 50, whereas in the LADA group, only 21.7% were under 50 at the time of diagnosis.

With respect to weight, patients with LADA had a significantly greater mean BMI (29.5 kg/m^2^) than non–LADA AD patients did (24.2 kg/m^2^). In addition, 68.8% of non–LADA AD patients had a normal body (<25 kg/m^2^), which is much greater than the 25.4% observed in patients with LADA. In contrast, obesity (BMI > 30 kg/m^2^) was much more prevalent in the LADA group.

Differences were also found in the presentation of DM, as 87.5% of non–LADA AD patients had symptoms of hyperglycemia at diagnosis, compared with only 26.7% of people with LADA.

Furthermore, the severity of hyperglycemia was greater in non-LADA AD patients, with much higher HbA1c levels at the time of diagnosis (10.6% vs. 7.8% in LADA patients).

With regard to cardiometabolic risk, patients with non–LADA autoimmune diabetes had a more favorable lipid profile, with higher HDL cholesterol levels (60.1 vs. 50.3 mg/dL) and significantly lower triglyceride levels (87 vs. 123.8 mg/dL) than patients with LADA did. In addition, two of the three metabolic indices studied (Triglycerides/HDL index and Triglycerides/glucose/BMI index) were notably higher in patients with LADA.

Among cardiovascular conditions, high blood pressure was much more common in the LADA group (41.7%) than in the non–LADA autoimmune group (12.5%), although no statistically significant differences were found in hypercholesterolaemia, chronic kidney disease, or a history of autoimmune diseases (personal or familial) between the two groups of antibody-positive patients.

With respect to the anti-pancreatic islet antibodies studied, the most common in both LADA patients and non-LADA AD patients was anti-GAD 65 (85% and 93%, respectively). The presence of IA-2 antibodies was more prevalent in non-LADA autoimmune diabetes patients (50% versus 35.2%, although the difference was not statistically significant).

The simultaneous presence of both antibodies was more common in patients with non-LADA autoimmune diabetes than in those with LADA (43.8% versus 16.7%).

When the prevalence of antithyroid antibodies was compared between the two groups with autoimmune diabetes, both antibodies (TPO-Ab and Tg-Ab) were more common in patients with non-LADA AD, but only TPO-Ab was significantly different.

## 4. Discussion

The results of the descriptive study conducted by our group, which was based on 1006 adult patients with a recent diagnosis of diabetes at primary care centers in Madrid, revealed a prevalence of autoimmune diabetes of 7.56%. This percentage falls within the range of 2% to 12% reported in various populations and reinforces the need for accurate classification at the time of diagnosis. In the recommendations on “Diagnosis and Classification of Diabetes: Standards of Care in Diabetes, 2026,” it is emphasized that both T1DM and T2DM are heterogeneous diseases whose presentation can vary considerably and that classification errors are common, with up to 40% of adults with T1DM initially misclassified as having T2DM [11].

In our study, we also observed that the subgroup of patients with autoimmunity was not homogeneous, as 21.05% of them were insulin-dependent from the outset, highlighting that autoimmunity in adults with diabetes is not always “latent”.

Although the patients with AD in our sample were significantly thinner than those with T2DM were, their mean BMI was 28.3 kg/m^2^, indicating that a large proportion of them were overweight and that up to 33% were obese. This finding is important and is consistent with the ADA’s recommendations, which explicitly state that obesity should not preclude testing for autoimmunity if other factors suggest the presence of AD [11]. In this regard, most comparative studies between AD and T2DM tend to find a lower BMI in patients with AD [12,13,14,15,16]; however, healthcare professionals should not base a clinical suspicion of AD solely on a low BMI, as this would result in a large number of undiagnosed cases in primary care settings.

Compared with patients with T2DM, people with LADA typically have lower levels of insulin resistance and a greater degree of autoimmune β-cell dysfunction, resulting in a phenotype intermediate between classic T1DM and T2DM. Consequently, they tend to be leaner than patients with T2DM, although their BMI is typically higher than that observed in patients with classic type 1 diabetes.

In our study, this gradient was also evident when comparing patients with LADA to those with non-LADA AD (Table 2). A BMI < 25 kg/m^2^ was significantly more common among patients with non-LADA autoimmune diabetes (68.8%) than among those with LADA (25.4%; *p* = 0.013). These findings support the idea that a more pronounced autoimmune component is typically associated with a leaner phenotype at the time of diagnosis, although overweight and obesity may also be present in patients with autoimmune diabetes.

A young age at the time of diagnosis is another factor that could suggest the presence of autoimmunity, as demonstrated in our study (27.6% of patients with autoimmunity were under 50 years of age at the time of their DM diagnosis, compared with only 14.5% of those with T2DM). However, advanced age does not rule out the disease, as 25% of patients with AD in our sample were at least 70 years old. With respect to age, most studies agree that there is a higher incidence of AD among younger adults [13,15], although others do not find such an association [17].

Patients with AD in our study had a more favorable metabolic and cardiovascular risk profile despite having a significantly higher HbA1c level at diagnosis (8.4% vs. 7.7% in patients with T2DM). They had higher HDL cholesterol levels and lower triglyceride levels (116 vs. 146.7 mg/dL). These findings are consistent with those of other studies [13,14], in which AD is associated with a more favorable lipid profile and a lower incidence of metabolic syndrome than conventional T2DM is. Furthermore, the three metabolic indices studied were lower in patients with AD than in patients without autoimmunity, suggesting lower insulin resistance in the AD group.

The prevalence of hyperglycemic symptoms at diagnosis (polyuria, polydipsia, and weight loss) was much greater in patients with autoimmune diabetes (39.5%), although a significant percentage (17.3%) of patients with T2DM also exhibited these symptoms.

Our study revealed a significantly greater prevalence of other autoimmune diseases in patients with AD than in those with T2DM (17.1% vs. 6.7%), suggesting that this history should be considered during medical history interviews at the onset of the disease. Furthermore, the prevalence of thyroid autoimmunity in patients with AD is greater than that in patients with T2DM, as other studies have already shown. Table S1 shows the prevalence and pairwise comparisons of personal and family histories of autoimmune diseases according to diabetes phenotypes, indicating, in general, that such personal and family histories of autoimmunity are more common in autoimmune diabetes—and, in particular, in non-LADA autoimmune diabetes.

With respect to the subanalysis conducted in patients with autoimmune diabetes, it is noteworthy that a significant percentage of patients (21.05%) were insulin-dependent within the first 6 months after diagnosis and were therefore classified as having AD without LADA criteria. This group of patients behaves more similarly to T1DM patients, and most of the distinguishing characteristics between autoimmune diabetes and T2DM are much more pronounced.

In our study, the simultaneous presence of the two autoantibodies analyzed (anti-GAD65 and anti-IA-2) was significantly more common in patients with non-LADA autoimmune diabetes than in those with LADA (43.8% versus 16.7%, respectively; *p* = 0.021). This finding is consistent with the existing literature and supports the idea that a higher degree of autoimmunity may be associated with a more aggressive course of the disease, characterized by faster β-cell deterioration and earlier insulin dependence.

However, since β-cell function and C-peptide levels were not measured in our study, we cannot directly assess the relationship between autoantibody burden and the rate of β-cell deterioration in our cohort.

Early diagnosis has direct therapeutic implications according to the 2026 ADA Standards [11]. For patients with AD, insulin therapy is essential for preventing ketosis and tissue catabolism, although the timing of its initiation varies depending on the patient and their insulin reserve. Identifying autoimmunity in primary care would help avoid “therapeutic inertia” and accelerate the initiation of insulin therapy before severe deterioration or ketoacidosis occurs. The timing of the initiation of insulin therapy in primary care may be influenced by the healthcare provider’s behavior and by delayed diagnosis. This classification variable may be partially biased by patterns of clinical practice, rather than reflecting solely the underlying beta-cell reserve.

In a primary care study involving 3031 patients with type 2 diabetes who were tested for anti-GAD-65 antibodies, primary care physicians’ knowledge of autoimmune diabetes influenced the initiation of insulin therapy [18].

In summary, a low or normal BMI is one of the clinical features that may raise suspicion of autoimmune diabetes (LADA or non-LADA AD); however, BMI alone is not sufficient to identify patients who should be screened for islet autoantibodies. In fact, type 1 diabetes, especially LADA, can occur across a wide range of BMI values, even in overweight or obese individuals.

The likelihood of having AD increases when a low BMI is present along with other clinical features suggestive of autoimmunity. Fourlanos et al. identified five key clinical features associated with LADA: age at onset < 50 years, acute hyperglycemic symptoms, BMI < 25 kg/m^2^, personal history of autoimmune disease, and family history of autoimmune disease. The presence of at least two of these characteristics (LADA clinical risk score ≥ 2) yielded a sensitivity of 90% and a specificity of 71% for identifying LADA, while a score ≤ 1 had a negative predictive value of 99% [12].

Therefore, rather than applying a specific BMI threshold as a standalone criterion, we believe that autoantibody testing should be considered in patients with suspected newly diagnosed T2DM who present with a combination of clinical features suggestive of AD. This approach is likely to be more clinically useful and more cost-effective in the primary care setting.

We believe that there is significant room for improvement in the diagnosis and treatment of autoimmune diabetes in primary care, a condition that remains underdiagnosed and inadequately managed according to current guidelines.

Our study, conducted in the primary care setting, confirms and supports this reality, which can and must be improved.

## 5. Limitations and Strengths

This study has several limitations that should be considered when interpreting the findings. First, its cross-sectional design precludes establishing temporal or causal relationships between autoimmune status and clinical or metabolic characteristics. Second, although recruitment was multicentric, the sample was obtained through consecutive/convenience sampling, which may introduce selection bias and limit generalizability beyond the participating primary care centers. Third, some variables, particularly lifestyle habits and self-perceived health, rely on patient self-reports and may be subject to recall or social desirability bias. Fourth, although pancreatic islet autoantibodies were measured using standardized laboratory procedures, only two autoantibodies (anti-GAD65 and IA-2) were available in routine practice; therefore, some cases of autoimmune diabetes with other antibody profiles may have been missed. Fifth, steroid-induced diabetes was not an exclusion criterion in this study, although only 3.0% of the patients were receiving corticosteroids at the time of enrollment. Sixth, a limitation of this study is the limited ethnic diversity of the study population, which consisted predominantly of people of European descent. Therefore, the results may not be fully generalizable to populations of different ethnic origins. Furthermore, socioeconomic status was not assessed, which prevented an evaluation of its potential influence on the prevalence, diagnosis, and clinical presentation of autoimmune diabetes in adults.

Another consideration to keep in mind as a potential limitation of this study is that C-peptide levels were not measured in the participants. The study protocol was developed in 2018, and funding was awarded in 2019 through the Carlos III Health Institute (ISCIII) (PI19/01569). At that time, the primary objective was to characterize autoimmune diabetes in adults in the primary care setting and to validate or improve clinical tools such as the Fourlanos score. Consequently, the diagnosis of LADA was based on the Immunology of Diabetes Society (IDS) criteria and the presence of autoantibodies against pancreatic islets, which were the standard diagnostic markers used in most epidemiological studies. Current recommendations from international experts primarily use C-peptide to guide therapeutic decisions, as lower C-peptide levels are associated with more advanced β-cell dysfunction and an earlier need for insulin treatment.

There are also some methodological limitations. First, numerous statistical comparisons were performed, particularly in Table 1 and Table 2. Given the exploratory nature of these analyses, no formal adjustment for multiple testing was applied, which increases the risk of Type I error. Consequently, results with *p*-values close to the conventional threshold for statistical significance should be interpreted with caution. Second, the subgroup of autoimmune diabetes other than LADA included only 16 patients. Although statistically significant differences were identified, the small sample size limits the precision of the estimates—as reflected by the 95% confidence intervals provided—and increases the possibility that some results may be unstable. Therefore, these results should be considered hypothesis-generating rather than confirmatory and require validation in larger, independent cohorts. Third, missing data for certain variables required the use of effective denominators (N′), which may reduce precision in subgroup comparisons.

Despite these limitations, the study has notable strengths. It represents one of the largest real-world assessments of adult-onset autoimmune diabetes conducted in primary care in Spain, using standardized laboratory measurements and harmonized data collection across 12 centers. The inclusion of detailed clinical, metabolic, lifestyle, and cardiometabolic indices provides a comprehensive characterization of autoimmune and non-autoimmune diabetes phenotypes at diagnosis. Importantly, this study addresses a critical diagnostic gap in routine primary care, offering empirical evidence that may support more accurate classification and earlier therapeutic decision-making in adults with newly diagnosed diabetes.

## 6. Conclusions

Our study in Madrid demonstrated that autoimmune diabetes is a common condition in primary care that must be considered in adult patients with newly diagnosed diabetes who should be diagnosed early. Clinical suspicion may be based on the set of characteristics most commonly associated with this disease rather than only on a normal or low BMI or elevated HbA1c levels at diagnosis. The combination of several of these characteristics, such as a very high HbA1c level, a low BMI, a more favorable lipid profile, a young age at diagnosis, hyperglycemic symptoms at the onset of the disease and a history of autoimmunity, should alert the primary care physician to the possible presence of AD, prompting them to order antibody and C-peptide testing.

It would be advisable to develop validated risk scales for the disease, which can be systematically applied in primary care to facilitate a correct and earlier diagnosis of AD and make the ordering of anti-pancreatic islet antibodies cost-effective, in line with the personalized medicine framework promoted by current international standards.

## Figures and Tables

**Table 1:** Characteristics of Newly Diagnosed Diabetes Patients.

Characteristic Value	Antibody-Positive (*N* = 76)	Antibody-Negative (*N* = 930)	*p* Value
Age (years)			
Mean (*SD*)	59.2 (12.3)	62.6 (10.6)	0.020 ¥
<60 years, *n* (%)	37 (48.7)	367 (39.5)	0.196 *
60–69 years, *n* (%)	20 (26.3)	332 (35.7)	
≥70 years, *n* (%)	19 (25)	231 (24.8)	
Age at diagnosis			
<50 years, *n* (%)	21 (27.6)	135 (14.5)	0.002 *
Male sex, n (%)	39 (51.3)	524 (56.3)	0.396 *
Educational level			
Low-medium, *n* (%)	24 (31.6)	338 (36.4)	0.402 *
High, *n* (%)	52 (68.4)	591 (63.6)	
BMI			
Mean (*SD*)	28.3 (6.1)	30.9 (5.6)	0.001 ¥
<25 kg/m^2^, *n* (%)	26 (34.7)	108 (11.7)	<0.001 *
25–29 kg/m^2^, *n* (%)	19 (25.3)	345 (37.3)	
30–39 kg/m^2^, *n* (%)	25 (33.3)	413 (44.6)	
≥40 kg/m^2^, *n* (%)	5 (7.6)	59 (6.4)	
Hyperglycemic symptoms at diagnosis, *n (%)*	30 (39.5)	161 (17.3)	<0.001 *
Physical activity			
Low, *n/N′* (%)	32/75 (42.7)	486/924 (52.6)	0.098 *
Mediterranean diet screener (MEDAS)			
Low adherence (MEDAS ≤ 5), *n*/*N′* (%)	2/75(2.7)	71/924 (7.7)	0.226 *
Medium adherence (MEDAS 6–10), *n*/*N′* (%)	50/75 (66.7)	613/924 (66.3)	
High adherence (MEDAS 11–14), *n*/*N′* (%)	23/75 (30.7)	240/924 (26)	
Self-perceived health status			
Poor, *n/N′* (%)	5/76 (6.6)	15/926 (1.6)	0.004 *
Fair, *n/N*’ (%)	15/76 (19.7)	204/926 (22)	
Good, *n/N′* (%)	29/76 (38.2)	472/926 (51)	
Very good, *n/N′* (%)	25/76 (32.9)	200/926 (21.6)	
Excellent, *n/N′* (%)	2/76 (2.6)	35/926 (3.8)	
HbA1c at diagnosis (%), *mean (SD)*	8.4 (2.6)	7.7 (1.9)	0.015 ¥
HbA1c last 6 months (%), *mean (SD)*	6.8 (1.2)	6.6 (1.0)	0.136 ¥
Variation between both HbA1c, *mean (SD)*	−0.9 (0.3)	−0.9 (0,2)	0.314 ¥
Antithyroid antibodies			
TPO-Ab, *n* (%)	24 (31.6)	74 (8.0)	<0.001 *
Tg-Ab, *n*/*N′* (%)	15/76 (19.7)	72/925 (7.8)	<0.001 *
Cholesterol total, mg/dL, *mean (SD)*	180.7 (34.3)	173.5 (38.7)	0.001 ¥
LDL Cholesterol, mg/dL, *mean (SD)*	106.1 (32.4)	98.3 (34.4)	0.055 ¥
HDL Cholesterol, mg/dL, *mean (SD)*	52.4 (12.6)	47.5 (13.0)	0.001 ¥
Triglycerides, mg/dL, *mean (SD)*	116 (59.2)	146.7 (99.5)	0.008 ¥
Triglycerides/HDL index, *mean (SD)*	2.48 (1.6)	3.47 (3.1)	<0.001 ¥
Triglycerides/glucose index, *mean (SD)*	8.93 (0.6)	9.07 (0.6)	0.047 ¥
Triglycerides/glucose/BMI index, *mean (SD)*	258.7 (57.7)	285.1 (57.8)	<0.001 ¥
Arterial hypertension, *n*/*N′* (%)	27/76 (35.5)	532/927 (57.4)	<0.001 *
Hypercholesterolemia, *n*/*N′* (%)	33/76 (43.4)	622/927 (67.1)	<0.001 *
Hypertriglyceridemia, *n*/*N′* (%)	13/76 (17.1)	251/927 (27.1)	0.058 *
Coronary artery disease, *n*/*N′* (%)	1/76 (1.3)	73/927 (7.9)	0.035 *
Cerebrovascular disease, *n*/*N′* (%)	2/76 (2.6)	23/927 (2.5)	0.936 *
Peripheral artery disease, *n*/*N′* (%)	1/76 (1.3)	30/927 (3.2)	0.352 *
Heart failure, *n* (%)	0 (0)	22 (2.4)	0.174 *
Atrial fibrillation, *n*/*N′* (%)	3/76 (3.9)	38/927 (4.1)	0.949 *
Metabolic syndrome, *n*/*N′* (%)	29/75 (38.7)	560/927 (60.4)	<0.001 *
Renal status, *n* (%)			
Normal	67 (88.2)	822 (88.4)	0.858 *
eGFR < 60 mL/min/1.73 m^2^	2 (2.6)	26 (2.8)	
Microalbuminuria	6 (7.9)	58 (6.2)	
Microalbuminuria & eGFR < 60	1 (1.3)	24 (2.6)	
Family history of Type 1 diabetes mellitus, *n/N′* (%)	8/67 (11.9)	62/844 (7.3)	0.174 *
Family history of Type 2 diabetes mellitus, *n/N′* (%)	37/70 (52.9)	539/864 (62.4)	0.115 *
Family autoimmune disease, *n*/*N′* (%)	11/76 (14.5)	80/927 (8.6)	0.088 *
Personal autoimmune disease, *n*/*N′* (%)	13/76 (17.1)	62/927 (6.7)	0.001 *

* Chi-square test. ¥ Student’s *t*-test for comparing means between two groups. *N′* corresponds to the number of individuals with no missing data for each variable and therefore represents the effective denominator for percentage calculations. BMI, body mass index; TPO-Ab, thyroid peroxidase antibody; Tg-Ab, thyroglobulin antibodies.

**Table 2:** Clinical and Biochemical Characteristics of Patients with Newly Diagnosed Autoimmune Diabetes, According to LADA Status.

	Antibodies Positives & LADA (*N*= 60)	Antibodies Positives with Non-LADA AD (*N* = 16)	*p*-Value
Age (years)			
mean (*SD*; 95% CI)	61.8 (11.4; 58.9–64.7)	51.7 (12.5; 45.0–58.4)	0.004
median (IQR)	62.4 (18.4)	52.2 (16.4)	
<60 years, *n* (%; 95% CI)	24 (40.0; 27.6–52.4)	13 (81.3; 62.2–100.0)	0.012 *
60–69 years, *n* (%; 95% CI)	19 (31.7; 19.9–43.5)	1 (6.3; 0.0–18.2)	
≥70 years, *n* (%; 95% CI)	17 (28.3; 16.9–39.7)	2 (12.5; 0.0–28.7)	
Age at diagnosis			
<50 years, *n* (%; 95% CI)	13 (21.7; 11.3–32.1)	8 (50.0; 25.5–74.5)	0.024
Male sex, *n* (%; 95% CI)	32 (53.3; 40.7–65.9)	7 (43.8; 19.5–68.1)	0.496
Educational level			
Low-medium, *n* (%; 95% CI)	20 (33.3; 21.4–45.2)	4 (25.0; 3.8–46.2)	0.524
High, *n* (%; 95% CI)	40 (66.7; 54.7–78.5)	12 (75.0; 53.8–96.2)	
BMI			
mean (*SD*; 95% CI)	29.5 (6.2); (27.9–31.1)	24.2 (3.6); (22.3–26.2)	0.001
median (IQR)	29.8 (8.9)	23.1 (5.6)	
<25 kg/m^2^, *n* (%; 95% CI)	15 (25.4; 14.4–36.4)	11 (68.8; 46.1–91.5)	0.013 *
25–29 kg/m^2^, *n* (%; 95% CI)	15 (25.4; 14.4–36.4)	4 (25.0; 3.8–46.2)	
30–39 kg/m^2^, *n* (%; 95% CI)	24 (40.7; 28.3–53.1)	1 (6.3; 0.0–18.2)	
≥40 kg/m^2^, *n* (%; 95% CI)	5 (8.5; 1.4–15.6)	0 (0.0; 0.0–0.0)	
Hyperglycemic symptoms at diagnosis, *n* (%; 95% CI)	16 (26.7; 15.5–37.9)	14 (87.5; 71.3–100.0)	<0.001
Physical activity			
Low, *n/N′* (%; 95% CI)	27 (45.8; 33.2–58.4)	5 (31.3; 8.6–54.0)	0.298
Mediterranean diet screener (MEDAS)			
Low adherence (MEDAS ≤ 5), *n/N′* (%; 95% CI)	1/59 (1.7; 0.0–5.0)	1/16 (6.3; 0.0–18.2)	0.445
Medium adherence (MEDAS 6–10), *n/N′* (%; 95% CI)	41/59 (69.5; 57.8–81.3)	9/16 (56.2; 31.9–80.5.2)	
High adherence (MEDAS 11–14), *n/N′* (%; 95% CI)	17/59 (28.8; 17.3–40.4)	6/16 (37.5; 13.8–61.2)	
Self-perceived health status			
Poor, *n* (%; 95% CI)	5 (8.3; 1.3–15.3)	0 (0.0; 0.0–0.0)	0.198
Fair, *n* (%; 95% CI)	14 (23.3; 12.6–34.0)	1 (6.3; 0.0–18.2)	
Good, *n* (%; 95% CI)	23 (38.3; 26.6–50.6)	6 (37.5; 13.8–61.2)	
Very good, *n* (%; 95% CI)	17 (28.3; 16.9–39.7)	8 (50.0; 25.5–74.5)	
Excellent, *n* (%; 95% CI)	1 (1.7; 0.0–5.0)	1 (6.3; 0.0–18.2)	
HbA1c at diagnosis (%)			
mean (*SD*; 95% CI)	7.8 (2.2; 7.2–8.4)	10.6 (2.9; 9.1–12.5)	0.001
median (IQR)	7.1 (2.2)	11.4 (4.8)	
HbA1c last 6 months (%)			
mean (*SD*; 95% CI)	6.8 (1.2; 6.5–7.1)	6.6 (1.1; 6–0−7.2)	0.495
median (IQR)	6.7 (1.3)	6.4 (0.8)	
Variation between both HbA1c			
mean (*SD*; 95% CI)	−0.92 (0.2: −0.9–1.0)	−0.68 (0.2; −0.6–0.8)	0.001
median (IQR)	−0.92 (0.23)	−0.57 (0.36)	
Antithyroid antibodies			
TPO-Ab, *n* (%; 95% CI)	15 (25; 14.0–36.0)	9 (56.3; 32.0–80.6)	0.017
Tg-Ab, *n/N′* (%; 95% CI)	10 (16.7; 7.3–26.1)	5 (31.3; 8.6–54.0)	0.193
Anti-islet antibodies			
GADA-65, *n* (%; 95% CI)	51 (85: 76.0–94.0)	15 (93.8;82.0–100.0)	0.358
IA-2, *n/N′* (%; 95% CI)	19/54 (35.2; 22.5–47.9)	8/16 (50.0; 25.5–74.5)	0.285
GADA-65 and IA-2, *n* (%; 95% CI)	10 (16.7; 7.3–26.1)	7 (43.8; 19.5–68.1)	0.021
Cholesterol total, mg/dL			
mean (*SD*; 95% CI)	179.3 (35; 170.3–188.3)	186.2 (32; 169.1–203.3)	0.491
median (IQR)	179.0 (52)	185.0 (53)	
HDL Cholesterol, mg/dL			
mean (*SD;* 95% CI)	50.3 (11; 47.5–53.1)	60.1 (14; 52.6–67.6)	0.014
median (IQR)	48.5 (13)	65.0 (27)	
LDL Cholesterol, mg/dL			
mean (*SD*; 95% CI)	105.6 (33; 97.1–114.1)	107.9 (29; 92.5–123.4)	0.740
median (IQR)	103.5 (51)	120.0 (53)	
Triglycerides, mg/dL			
mean (*SD*; 95% CI)	123.8 (62; 107.8–139.8)	87 (37; 67.2–106.7)	0.024
median (IQR)	108.0 (94)	82.0 (48)	
Triglycerides/HDL index			
mean (*SD*; 95% CI)	2.7 (1.7; 2.3–3.1)	1.6 (1.0; 1.1–2.1)	0.007
median (IQR)	2.4 (2.3)	1.4 (1.4)	
Triglycerides/glucose index			
mean (*SD*; 95% CI)	9.0 (0.6; 8.8–9.2)	8.9 (0.5; 8.6–9.2)	0.656
median (IQR)	8.9 (0.9)	9.0 (0.8)	
Triglycerides/glucose/BMI index			
mean (*SD*; 95% CI)	268 (59; 252.8–283.2)	225 (38; 204.8–245.2)	0.007
median (IQR)	275 (84.1)	217 (66.8)	
Arterial hypertension, *n* (%; 95% CI)	25 (41.7; 29.2–54.2)	2 (12.5; 0.0–28.7)	0.030
Hypercholesterolemia, *n* (%; 95% CI)	27 (45.0; 32.4–57.6)	6 (37.5; 13.8–61.2)	0.591
Hypertriglyceridemia, *n* (%; 95% CI)	11 (18.3; 8.5–28.1)	2 (12.5; 0.0–28−7)	0.582
Chronic kidney disease, *n* (%; 95% CI)	7 (11,7; 3.6–19.8)	2 (12.5; 0.0–28.7)	0.927
Coronary artery disease, *n* (%; 95% CI)	1 (1.7; 0.0–5.0)	0 (0.0; 0.0–0.0)	0.603
Cerebrovascular disease, *n* (%; 95% CI)	2 (3.3; 0.0–7.8)	0 (0.0; 0.0–0.0)	0.459
Peripheral artery disease, *n* (%; 95% CI)	1 (1.7; 0.0–5.0)	0 (0.0; 0.0–0.0)	0.603
Atrial fibrillation, *n* (%; 95% CI)	2 (3.3; 0.0–7.8)	1 (6.3; 0.0–18.2)	0.594
Metabolic syndrome, *n/N′* (%; 95% CI)	26/59 (44.1; 31.5–56.7)	3/16 (18.8; 0.0–37.9)	0.065
Family history of Type 1 diabetes mellitus, *n/N′* (%; 95% CI)	6/51 (11.8; 3.0–20.7)	2/16 (12.5; 0.0–28.7)	0.937
Family history of Type 2 diabetes mellitus, *n/N′* (%; 95% CI)	31/54 (57.4; 44.2–70.6)	6/16 (37.5; 13.8–61.2)	0.161
Family autoimmune disease, *n* (%; 95% CI)	5 (8.3; 1.3–15.3)	6 (37.5; 13.8.0–61.2)	0.003
Personal history of autoimmune disease, *n* (%; 95% CI)	10 (16.7; 7.3–26.1)	3 (18.8; 0.0–37.9)	0.844

Continuous variables are presented as mean (standard deviation [SD]) and/or median (interquartile range [IQR]). Categorical variables are presented as *n* (%). Between-group comparisons were performed using the Mann–Whitney U test for continuous variables and the chi-square test or Fisher’s exact test for categorical variables, as appropriate. * Ordinal categorical variables with a linear trend were analyzed using the linear-by-linear association test. Percentages were calculated using the number of participants with available data for each variable. *N′* corresponds to the number of individuals with no missing data for each variable and therefore represents the effective denominator for percentage calculations. BMI, body mass index; HbA1c, glycated hemoglobin; LADA, latent autoimmune diabetes in adults; MEDAS, Mediterranean Diet Adherence Screener; TPO-Ab, thyroid peroxidase antibodies; Tg-Ab, thyroglobulin antibodies.

## Data Availability

There are restrictions on data availability for the LADA-PC dataset because of the signed consent agreements around data sharing, which only allow access to external researchers for research following the project’s purposes once it has been completed. The requestors wishing to access the LADA-PC data used in this study can request it to the LADA-PC Steering Committee: lada.ap.research@gmail.com.

## Supplementary Materials

The following supporting information can be downloaded at: https://www.mdpi.com/article/10.3390/jcm15218317/s1, Table S1: Prevalence and pairwise comparisons of personal and family history of autoimmune disease across diabetes phenotypes.
