# Supplementary material for: Toward an Appropriate Classification of Adult-Onset Diabetes Mellitus: A Descriptive Study in Primary Care
Source: J Clin Med. 2026 Oct 26;15(21):8317. doi: 10.3390/jcm15218317 (PMC13764355; doi:10.3390/jcm15218317)
Supplement: Supplementary file 1 [file jcm-15-08317-s001.zip › jcm-4607108-supplementary.pdf]

Supplementary Table S1. Prevalence and pairwise comparisons of personal and family history of autoimmune disease across diabetes phenotypes.

n	Phenotype	Personal History of Autoinmune Disease	Family Histoy of Autoinmune Disease
927	Non-autoinmune DM	6,70%	8,60%
16	Autoinmune DM without LADA	18,80%	37,50%
60	LADA	16,70%	8,30%
	p value*	**0.004**	**<0.001**
927	Non-autoinmune DM	6,70%	8,60%
16	Autoinmune DM without LADA	18,80%	37,50%
	p value†	0.15	**0.006**
927	Non-autoinmune DM	6,70%	8,60%
60	LADA	16,70%	8,30%
	p value†	**0.019**	0.935
16	Autoinmune DM without LADA	18,80%	37,50%
60	LADA	16,70%	8,30%
	p value*	0.844	**0.003**
76	Autoinmune DM	17,10%	14,50%
927	Non-autoinmune DM	6,70%	8,60%
	p value*	**0.001**	0.088

*Chi-square test; †Z-test for comparison of proportions.
